# FDM 3D-Printed Sustained-Release Gastric-Floating Verapamil Hydrochloride Formulations with Cylinder, Capsule and Hemisphere Shapes, and Low Infill Percentage

**DOI:** 10.3390/pharmaceutics14020281

**Published:** 2022-01-25

**Authors:** Haonan Qian, Di Chen, Xiangyu Xu, Rui Li, Guangrong Yan, Tianyuan Fan

**Affiliations:** 1 The State Key Laboratory of Natural and Biomimetic Drugs, School of Pharmaceutical Sciences, Peking University, Beijing 100191, China; 1610307327@pku.edu.cn (H.Q.); 1310307112@pku.edu.cn (D.C.); 1410307125@pku.edu.cn (R.L.); 2 School Beijing Key Laboratory of Molecular Pharmaceutics and New Drug Delivery Systems, School of Pharmaceutical Sciences, Peking University, Beijing 100191, China; 3 School of Mechanical Engineering and Automation, Beihang University, Beijing 100191, China; xyxu1990@buaa.edu.cn (X.X.); yangr@buaa.edu.cn (G.Y.)

**Keywords:** hot-melt extrusion, fused deposition modeling, 3D printing, floating systems, shape, infill percentage, sustained release

## Abstract

The aim of this work was to design and fabricate fused deposition modeling (FDM) 3D-printed sustained-release gastric-floating formulations with different shapes (cylinder, capsule and hemisphere) and infill percentages (0% and 15%), and to investigate the influence of shape and infill percentage on the properties of the printed formulations. Drug-loaded filaments containing HPMC, Soluplus^®^ and verapamil hydrochloride were prepared via hot-melt extrusion (HME) and then used to print the following gastric-floating formulations: cylinder-15, capsule-0, capsule-15, hemisphere-0 and hemisphere-15. The morphology of the filaments and the printed formulations were observed by scanning electron microscopy (SEM). The physical state of the drugs in the filaments and the printed formulations were characterized by X-ray diffraction (XRD), thermogravimetric analysis (TGA) and differential scanning calorimetry (DSC). The printed formulations were evaluated in vitro, including the weight variation, hardness, floating time, drug content and drug release. The results showed that the drug-loaded filament prepared was successful in printing the gastric floating formulations. Verapamil hydrochloride was proved thermally stable during HME and FDM, and in an amorphous state in the filament and the printed formulations. The shape and infill percentage of the printed formulations effected the hardness, floating time and in vitro drug release.

## 1. Introduction

Oral administration is a preferred drug delivery route for its high patient compliance, low cost, non-invasiveness and ease of use [1]. However, the bioavailability of conventional oral dosage forms may be influenced by short gastro-retentive time or unpredictable gastric emptying times, especially for drugs which are absorbed in the upper part of the digestive system, or more soluble in acidic environments [2]. To solve this problem, a series of gastro-retentive systems, including mucoadhesion systems, expandable systems and floating systems, were developed to prolong gastric residence time of drugs to obtain better drug absorption and bioavailability [3,4]. Among all these gastroretentive systems, the gastro-floating systems attracted special attention due to their low density that allowed them to float on gastric fluids without affecting the gastric emptying rate [4]. Traditionally, gastro-floating systems rely on swelling polymers, microporous components or gas-generating systems to achieve low density, which usually leads to their complex composition and difficulty in manufacturing [4,5]. In addition, the gastro-floating systems are sometimes restricted by floating lag time, which increases the risk of being excreted by the stomach before the dosage takes effect [6].

Since the first 3D-printed pharmaceutical product (Spritam^®^) was approved by the FDA in 2015 [7], the combination of 3D-printing technology and pharmaceutical science has become a new trend in pharmaceutical research. Taking advantage of the fabricated complex structure of 3D printing [8], gastro-floating systems have achieved many progresses. Gastric-floating tablets with low infill percentage were prepared based on fused deposition modeling (FDM) technology [9,10,11]. Semi-solid extrusion (SSE) 3D printing was also applied in developing gastro-floating systems with low density [12,13,14,15]. Moreover, tablet-in-device was designed and prepared for gastric-floating systems, which contain conventional or sustained-release tablets in FDM-printed floating devices [16,17]. Among these 3D printing gastric-floating systems, the reduction of tablet infill percentage is the most frequently adopted strategy, and the shape of the formulations were only cylinders or elliptic cylinders. As the geometrical shapes of 3D-printed formulations were reported to influence on the properties of formulations, such as the surface area, volume, weight, and in vitro drug release [18,19], we attempted to design and investigate 3D-printed gastric-floating systems with both low infill percentage (0% and 15%) and different geometrical shapes (cylinder, capsule and hemisphere) in this study.

Verapamil hydrochloride is a calcium channel blocker, which is commonly used to treat high blood pressure, angina, cardiac arrhythmias and cardiomyopathies [20]. The solubility of verapamil hydrochloride in hydrochloric acid solution (pH 1.0) and phosphate buffer solution (pH 6.8) is 360 mg/mL and 54 mg/mL, respectively [21]. The physicochemical properties of verapamil hydrochloride and its short half-life (4 h) make it a suitable candidate for preparing gastric-floating formulations [22]. Besides, the thermostability of verapamil hydrochloride provides a good opportunity for it to undergo hot-melt extrusion (HME) and FDM 3D printing at a high temperature [23].

In this study, FDM 3D-printing technology was adopted to prepare verapamil hydrochloride gastric-floating formulations. Cylinder-15, capsule-0, capsule-15, hemisphere-0 and hemisphere-15 (infill percentage at 0% or 15%) were designed and prepared. A series of in vitro characterizations were carried out to evaluate the drug-loaded filament and the 3D-printed gastric-floating formulations, including morphology, X-ray diffraction (XRD), thermogravimetric analysis (TGA), differential scanning calorimetry (DSC), weight variation, hardness, floating behavior, drug content, and in vitro drug release. In addition, the hardness of the formulation is tested in two orthogonal directions since the geometric shape and interior structure of the 3D-printed formulations is more complicated than conventional tablets.

## 2. Materials and Methods

### 2.1. Materials

Verapamil hydrochloride was purchased from Wuhan Yuancheng Gongchuang Technology Co., Ltd. (Wuhan, China). Commercial verapamil hydrochloride sustained-release tablets were purchased from Jiangsu Hengrui Medicine Co., Ltd. (Lianyungang, China). Hydroxypropyl methylcellulose (Affinisol^™^ HPMC HME 100lv) was donated by DOW Chemical Co., Ltd. (Shanghai, China). Polyvinyl caprolactam-polyvinyl acetate-polyethylene glycol copolymer (Soluplus^®^) was donated by BASF Co., Ltd. (Ludwigshafen, Germany). Polyethylene glycol (PEG 400) was purchased from Coolaber Technology Co., Ltd. (Beijing, China). Methanol (chromatographic grade) was purchased from ThermoFisher Co., Ltd. (Waltham, MA, USA). Other chemicals were of analytical grade and were used as received.

### 2.2. Preparation of Gastric-Floating Formulations

#### 2.2.1. Design of Formulations

**Figure 1:**
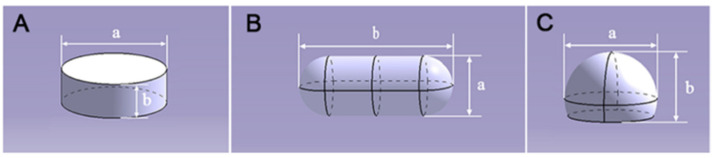
3D digital models (**A**) cylinder; (**B**) capsule; (**C**) hemisphere) of the FDM 3D-printed formulations.

The shapes of the 3D-printed gastric-floating formulations were designed to be cylinders, capsules and hemispheres, with 3D models as shown in Figure 1. The cylinder is the most commonly used shape for oral formulation, and the capsule and hemisphere are easily to float. Besides, all three shape formulations could be printed at low infill percentages. Moreover, the rectilinear grid infill pattern was chosen for the printed formulations, which could provide higher hardness when the infill percentage was low [24]. The infill percentage was set to be 0% for capsule-0 and hemisphere-0, and 15% for cylinder-15, capsule-15 and hemiphere-15. The digital model for printing the formulations was designed with AutoCAD 2014^®^ (Autodesk, San Rafael, CA, USA) and exported as a stereo lithography (.stl) file into the MakerWare software (v. 3.8.0.373, MakerBot, New York, NY, USA). The external dimension, layer height and shell number for the formulations were shown in Table 1.

#### 2.2.2. Preparation of Filaments

Filaments were prepared using the HME method. Beforehand, homogeneous mixing of the excipients and drug was obtained by the incremental method and thoroughly grinded with a mortar and pestle. To prepare the drug-loaded filaments, the verapamil hydrochloride, HPMC, Soluplus^®^ and PEG 400 were mixed homogeneously at a weight ratio of 10:42.5:42.5:5. The mixture was then extruded by a single-screw hot-melt extruder (FilaBot^®^ FOV1, USA) at 115 °C. The nozzle diameter of the extruder was 1.7 ± 0.1 mm. Blank filaments were also prepared by HME. The composition of blank filaments was HPMC, Soluplus^®^ and PEG 400 at a weight ratio of 47.2:47.2:5.6 (close to 42.5:42.5:5). The extruding condition was the same as that of the drug-loaded filaments.

#### 2.2.3. 3D Printing of Gastric-Floating Formulations

The drug-loaded filaments were used to fabricate the designed formulations by a desktop 3D printer (MakerBot Replicator 2X, MakerBot, New York, NY, USA). The temperatures for the nozzle and building platform were 195 °C and 90 °C, respectively. The speed of extruding and traveling for the printing head was 90–100 mm/s and 150 mm/s, respectively.

### 2.3. Morphology

Photographs of the blank filaments, drug-loaded filaments and 3D-printed formulations were taken with a digital camera (Canon IXUS 220 HS, Tokyo, Japan). The surface and section images of the blank filaments, drug-loaded filaments and 3D-printed formulations (at magnifications 30× and 1000×) were separately obtained by a scanning electron microscopy (SEM, JSM-IT300 Scanning Microscope, JEOL, Tokyo, Japan) at an accelerating voltage of 5.0 kV.

### 2.4. X-ray Powder Diffraction

X-ray powder diffraction (XRD, Mini Flex 600, Rigaku, Japan) was performed to assess the physical state of verapamil hydrochloride in the drug-loaded filaments and formulations. The analysis was carried out using a Cu X-ray source, and the operating current and voltage were set at 15 mA and 40 kV, respectively. All samples including verapamil hydrochloride, HPMC, Soluplus^®^, physical mixture (composed of verapamil hydrochloride, HPMC, Soluplus^®^ and PEG 400 at weight ratio of 10:42.5:42.5:5), drug-loaded filaments and 3D-printed formulations were scanned from 2θ of 5° to 60°at a speed of 5°/min [25,26,27].

### 2.5. Thermal Analysis

A thermogravimetric analyzer (Q600 SDT, TA Instruments, New Castle, DE, USA) was employed to assay the thermal stability of verapamil hydrochloride, HPMC, Soluplus^®^, physical mixture, drug-loaded filaments and 3D-printed formulations. The samples (about 5 mg) were weighted accurately, loaded in ceramic pans, and heated from room temperature to 500 °C. The test was carried out at a heating rate of 10 °C/min under a nitrogen purge of 100 mL/min. The data were analyzed using TA 2000 analysis software [28,29].

Differential scanning calorimetry (DSC, Q100 DSC, TA Instruments, USA) analysis was also conducted. After being dried in a desiccator, samples (about 5 mg) were loaded on aluminum pans, and the test was performed at a heating rate of 10 °C/min under a nitrogen purge of 50 mL/min. The heat scan from −20 to 200 °C was conducted for verapamil hydrochloride, HPMC, Soluplus^®^, physical mixture, drug-loaded filaments and 3D-printed formulations. Then, PEG 400 was scanned from −50 to 100 °C. Verapamil hydrochloride was assayed by a heat, quench and heat process. The data were analyzed using TA 2000 analysis software [30,31].

### 2.6. Weight

The 3D-printed formulations were weighed using an electronic analytical balance (ME104, Mettler Toledo, Zurich, Switzerland) (*n* = 10). The average weight and weight variances of the formulations were calculated, respectively.

### 2.7. Hardness

**Figure 2:**
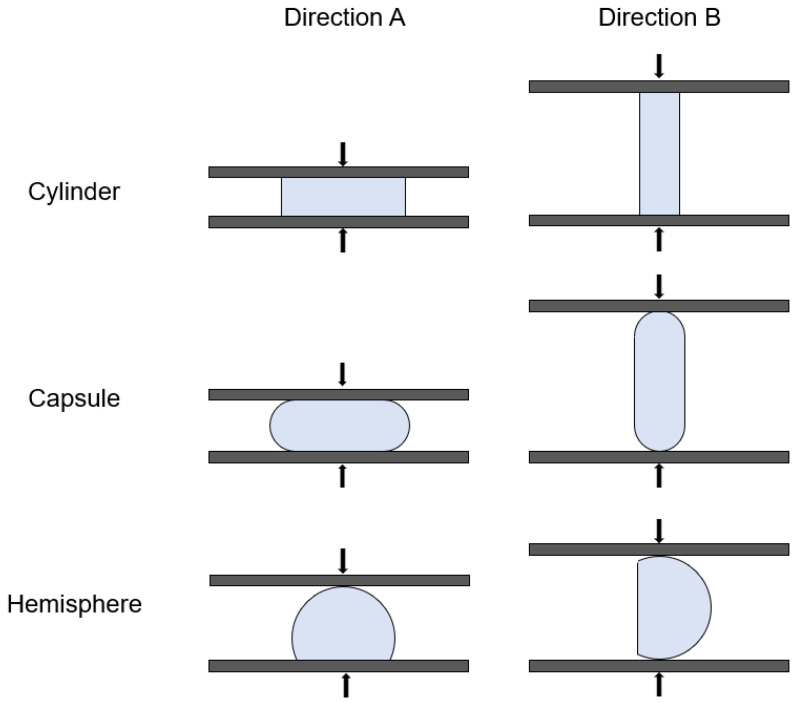
Illustration of the force applied to formulations in the hardness test.

The hardness of 3D-printed formulations was measured with a texture analyzer (MultiTest 2.5-I, Mecmesin, Slinfold, UK). A vertical pressure was put on the formulations by a 75 mm diameter probe at a speed of 1 mm/s and the maximum force of 800 N. The measurements were performed at two mutually perpendicular directions for each formulation (illustrated as Figure 2) (*n* = 6), considering that the structure of 3D-printed formulations was anisotropic, compared with that of traditional pressed formulations [10].

### 2.8. In Vitro Floating Behavior

The in vitro floating capacity of the formulations was tested according to the previous literatures [32,33]. Then, 100 mL of 0.1 mol/L hydrochloric acid solution was added in a beaker as floating medium and kept at 37 ± 0.5 °C in a thermostatic water bath. Each of the 3D-printed formulations was put into the medium and the floating behavior of the formulation was monitored and recorded. The tests were performed in triplicate for each formulation (*n* = 3).

### 2.9. Drug Content

Samples of the drug-loaded filaments and 3D-printed formulations (approximately 0.4 g) were precisely weighed and separately dissolved in 0.1 mol/L hydrochloric acid solutions (*n* = 3). After a proper dilution, the drug content of each sample was determined by high-performance liquid chromatography (HPLC).

Agilent UV-HPLC 1260 series (Agilent Technologies, Germany) were employed with Agilent Zorbax Extended C18 column (150 mm × 4.6 mm, particle size 5 µm) maintained at 40 °C. The mobile phase consisted of methanol and acetate buffer solution (pH was adjusted to 4.1 with triethylamine) at a volume ratio of 45:55. The flow rate of the mobile phase was 1.0 mL/min and the volume of sample injected was 20 μL. The analytical wavelength of the UV detector was set at 278 nm [21,22].

### 2.10. In Vitro Drug Release

The in vitro drug release of 3D-printed gastric-floating formulations was tested according to the United States Pharmacopeia (USP) dissolution apparatus I, and the commercial sustained-release verapamil hydrochloride tablet was used as a control (*n* = 6). 900 mL of 0.1 mol/L hydrochloric acid solution was added into each vessel and kept at 37.0 ± 0.5 °C with a stirring speed of 100 r/min [21,22]. 10 mL of the released medium was withdrawn at each predetermined time point, and replaced immediately with fresh medium at the same volume and temperature. All samples were filtered through a 0.2-μm filter and the concentrations of verapamil hydrochloride were assayed by HPLC at the same condition of drug content.

### 2.11. Statistical Analysis

In this study, *t*-test was employed using GraphPad Prism software (version 6.01) to analyze the results. Differences in results where *p* < 0.05 were considered significant, and *p* < 0.01 were considered extremely significant.

## 3. Results and Discussion

### 3.1. Morphology

**Figure 3:**
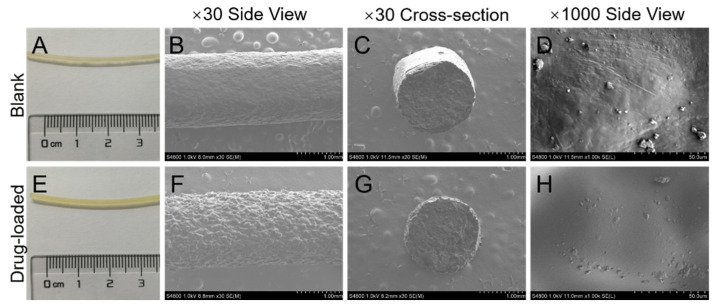
Photographs (**A**,**E**) and SEM (**B**–**D**,**F**–**H**) images of the blank and drug-loaded filaments.

The appearance and SEM photographs of the blank and drug-loaded filaments are shown in Figure 3. Both filaments showed smooth surface and homogeneous color (Figure 3A,E). The blank filaments were more transparent than the drug-loaded filaments. In the low magnification (30×) SEM side view, the blank filaments (Figure 3B) showed a smoother surface than drug-loaded filaments (Figure 3F), and both blank and drug-loaded filaments (Figure 3C,G) showed non-porous cross-sections. In the high magnification (1000×) side view, no crystal of the drug was observed on the surface of drug-loaded filaments (Figure 3H) compared with that of blank filaments (Figure 3D).

**Figure 4:**
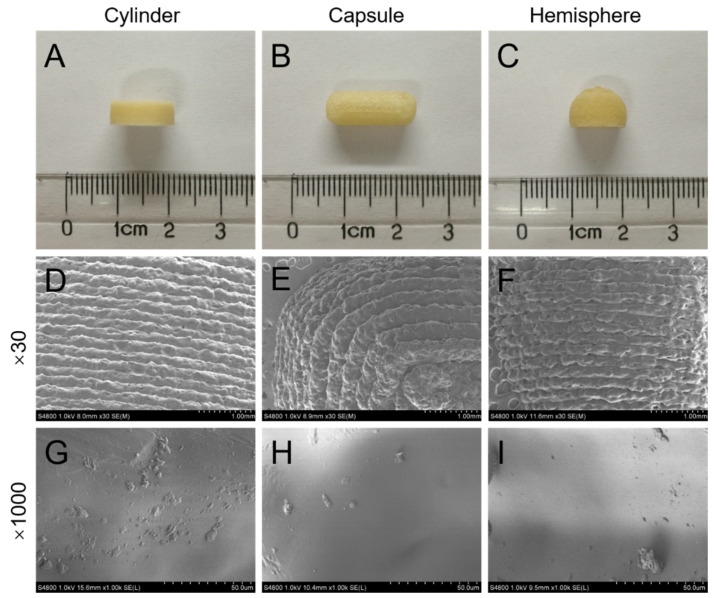
Photographs (**A**–**C**) and SEM (**D**–**I**) images of the cylinder, capsule and hemisphere formulations.

The appearance and SEM photographs of the cylinder-, capsule- and hemisphere-shaped formulations are shown in Figure 4. All the three shapes of formulations designed were successfully printed without printing defects (Figure 4A–C). SEM view at low magnification (30×) (Figure 4D–F) showed the tight layer-to-layer conjunctions in all three printed formulations, confirming good printing quality in the microstructure. No crystals of drug could be observed on the surface of all formulations in the SEM view at high magnification (1000×) (Figure 4G–I). As the temperature (195 °C) for printing formulations was higher than the melting point of verapamil hydrochloride (146 °C), the drug was supposed to disperse molecularly or in an amorphous state [23,34].

**Figure 5:**
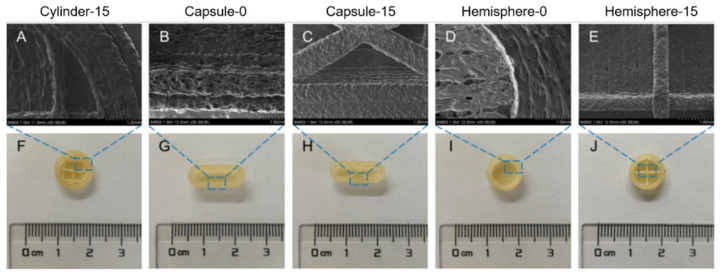
SEM section view (**A**–**E**) and corresponding photographs (**F**–**J**) of the formulations.

The appearance and SEM photographs of the cross-sections of the three formulations are further shown in Figure 5, presenting the inner structure of the printed formulations. Low infill percentage was designed for all formulations to ensure the feasibility of floating. When the infill percentage was set at 0% for cylinder formulations, the flat top tended to collapse, whereas capsule-0 and hemisphere-0 formulations were printed successfully by the effective support of arched structures. Thus, only 15% of the infill percentage was designed for the cylinder formulations (cylinder-15). In order to obtain comparable formulations among the three shapes of formulations, 15% of infill percentage was also utilized for capsule-15 and hemisphere-15.

### 3.2. X-ray Powder Diffraction

**Figure 6:**
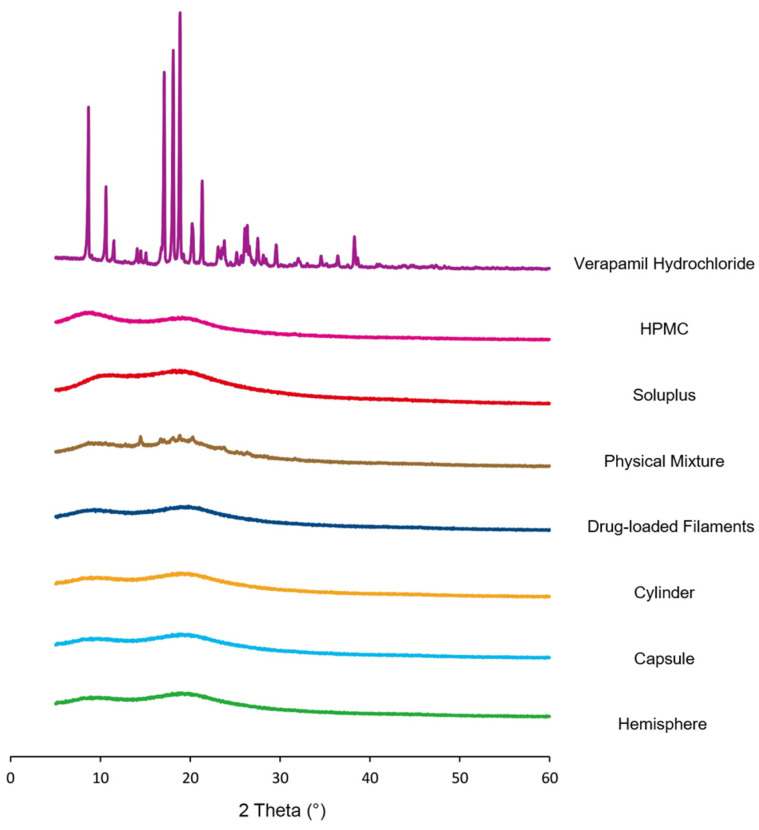
XRD results of verapamil hydrochloride, HPMC, Soluplus, physical mixture, drug-loaded filaments and formulations.

The result of XRD is shown in Figure 6. The pure verapamil hydrochloride showed several characteristic peaks at 2θ of 10.61°, 14.50°, 17.09°, 18.09°, 18.86°, 20.20°, 21.32°, 23.09°, 23.79°, and 26.35°, which was close to the literature reports that the prominent peaks from pure verapamil hydrochloride were observed at 2θ of 10.59°, 14.45°, 17.07°, 18.1°, 18.84°, 20.29°, 21.32°, 23.06°, 23.75°, and 26.29° [25,35]. The HPMC and Soluplus^®^ showed broad peaks with the peak of maximum intensity at 9.09° and 18.06°, respectively, which indicated their amorphous behavior. The diffraction peaks of verapamil hydrochloride could be found in the physical mixture but disappeared in the drug-loaded filaments and 3D-printing formulations, indicating that there was no drug crystal existing after HME and the 3D-printing process [36]. This was consistent with the SEM photograph in which no drug crystal was founded on either filament or formulation.

### 3.3. Thermal Analysis

**Figure 7:**
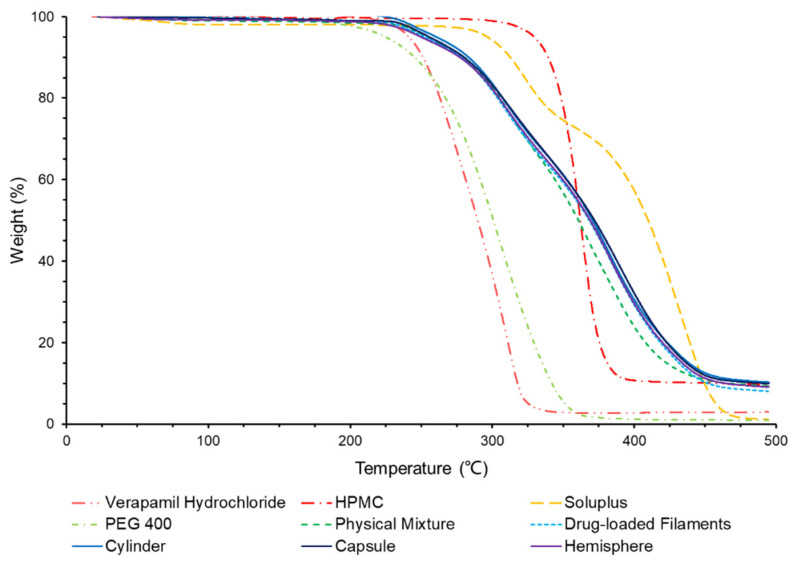
TGA results of verapamil hydrochloride, HPMC, Soluplus, physical mixture, drug-loaded filaments and formulations.

As shown in the Figure 7, the TGA result showed that the drug and excipients were all thermally stable at the temperature of HME (115 °C) and FDM printing (195 °C). All samples showed less than 5% weight loss at 195 °C, indicating their thermostability under FDM condition. The weight loss under 60 °C was considered as attributing to be the evaporation of water. The hygroscopicity of Soluplus^®^ and HPMC was in accordance with the previous reports [37,38,39].

**Figure 8:**
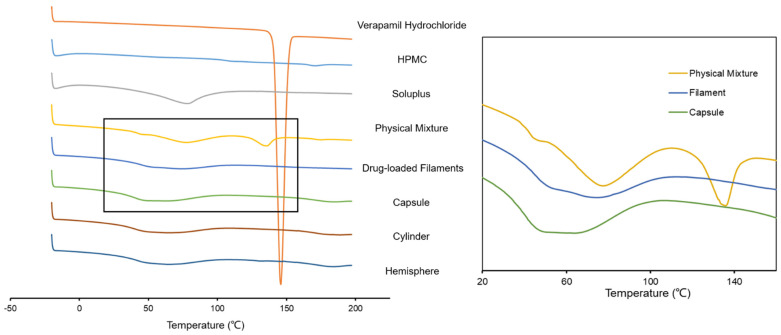
DSC results of verapamil hydrochloride, HPMC, Soluplus, physical mixture, drug-loaded filaments and formulations.

The DSC result is shown in Figure 8. An endothermic peak of verapamil hydrochloride showed a melting point at 145.9 °C, which was consistent with literature (138.5 °C~148.1 °C) [34]. An endothermic peak of physical mixture was also observed at 135.8 °C, indicating the existence of verapamil crystal. The decrease in melting point was supposed to be related with the plasticization of PEG 400. However, no obvious endothermic peak was observed in drug-loaded filaments or 3D-printing formulations, indicating that most of the drug did not exist in crystal form. The result was consistent with those of SEM and XRD. Combining the thermograms of verapamil hydrochloride (glass transition temperature (Tg) at 52.3 °C, Figure S1 in Supplementary Materials) and PEG 400 (melting point at 5 °C, Figure S2 in Supplementary Materials), the transitions of physical mixture, filament and formulations from 40 to 60 °C demonstrated that the amorphous drug existed and gradually became less in them. Still, the drug did not distribute molecularly homogenous in the formulations, which might have potential implications to the dissolution findings. Additionally, the Tg of pure Soluplus^®^ was at 78.4 °C, which was close to the previous report [40]. The Tg of Soluplus^®^ decreased successively in the order of physical mixture, filament and formulations, indicating the mixture was more homogeneous and the plasticization of PEG 400 was more effective.

### 3.4. Weight

The average weight of the 3D-printed gastric-floating formulations is shown in Table 1. The average weights of the cylinder-15 (373.5 ± 9.8 mg), capsule-0 (372.3 ± 17.5 mg) and hemisphere-0 (374.5 ± 18.8 mg) were close to each other (*p* > 0.05) and consistent with the model designed, which made it easier for evaluating the influence of external shapes on the floating and drug-release behavior of the formulations. For the capsule- or hemisphere-shaped formulations, those with lower infill percentage had smaller average weights. The average weights of capsule-0 and capsule-15 were 372.3 ± 17.5 mg and 404.5 ± 10.3 mg (*p* < 0.01), and the average weights of hemisohere-0 and hemisphere-15 were 374.5 ± 18.8 mg and 389.7 ± 10.1 mg (*p* < 0.05), respectively.

The relative standard deviation (RSD) of the formulation weight was calculated according to the standard deviation and average weight in Table 1. The data obtained were in the range of 2.62% to 5.02%. The RSD of the weight of 3D-printed formulations was supposed to reflect the printing precision and reproductivity in certain aspects [41]. For FDM 3D-printed formulations at average weight between 300 mg to 500 mg, the RSD of weight was reported from 0.31% to 8.17% [13,29,42,43,44,45,46], and the number of formulations reported for determining the weight variation was from 3 to 10 [29,42,47]. In this study, the RSD of weight (2.62~5.02%) and the number of formulations (*n* = 10) were both within the scope of literatures.

### 3.5. Hardness

The hardness of formulations is shown in Table 2. All formulations had sufficient hardness in the two testing directions (shown in Figure 2). The hardness of formulations was observingly affected by the tested direction, infill percentage and axial symmetry. Firstly, a decrease in hardness was observed more so in direction B than in direction A, especially for the cylinder- and hemisphere-shaped formulations. The result was supposed to be owing to the layer-by-layer structure of 3D-printing formulations, as the lateral force to layers in direction B broke the adhesion between layers more easily than vertical force to layers in direction A. Secondly, the formulations with higher infill percentage showed higher hardness, which could be found in the comparation of data between capsule-0 and capsule-15 or between hemisphere-0 and hemisphere-15 in both directions. The increase in infill percentage was considered to reinforce the mechanical strength of the formulations [48]. Thirdly, the symmetry of the formulation also affected its hardness behavior, especially when the force of the test direction was parallel to the layer. In the case of the cylinder- and capsule-shapes, the formulations were axial symmetric to the test force in both front and side view, and indeed exhibited good hardness. While the hardness of the hemisphere formulation significantly decreased where the shape was asymmetric in the side view. This phenomenon could be explained by the fact that the force added did not distribute evenly to the formulation, and broke it easily.

### 3.6. In Vitro Floating Behavior

**Figure 9:**
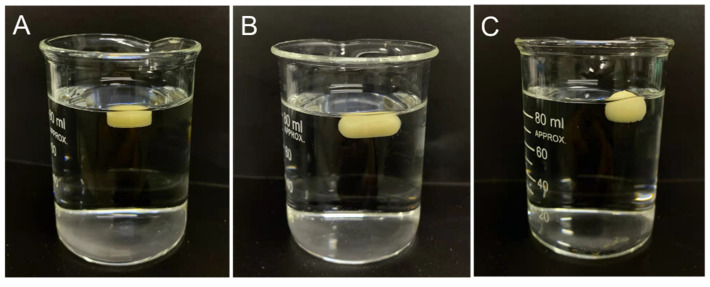
In vitro floating of the cylinder (**A**), capsule (**B**) and hemisphere (**C**) formulations at 0 h (without floating lag time).

The in vitro floating time of the 3D-printed formulations is shown in Table 2. There was no floating lag time observed in all the tests, as shown in Figure 9. The phenomenon was supposed owing to the density designed (0.638~0.827 g/mL, as shown in Table 1) which was lower than that of gastric juice (1.004 g/mL) [49]. The floating lag time was reported to raise the risk of gastric-floating formulations being expelled from the stomach before drug release [6]. Hence, the complicated work in traditional manufacturing processes, such as optimizing the formulation composition and the production process to avoid the floating lag time of gastric-floating formulations, was greatly reduced by the 3D-printing technology. On the other hand, as the infill percentage of the 3D-printed formulations was low in this study, the penetration of gastric fluid into the sealed center of formulation was easy, leading to the floating time being relatively short. A combinatory approach, such as a floating and expandable combinatory system, was suggested in a recent published literature which may help to overcome the limitation [50].

In this study, different external shapes of formulations were designed to investigate the influence of shape on the in vitro floating behavior. Though the theoretically calculated floating force of the hemisphere- or capsule-shaped formulations was larger than that of the cylinder-shaped formulations, the practical result revealed that the floating time of the hemisphere- and capsule-shaped formulations was shorter than that of the cylinder-shaped formulations (*p* < 0.05). During the in vitro floating test, the rupture of the hemisphere- and capsule-shaped formulations was observed faster than that of the cylinder-shaped formulations, which was considered to be owing to the less connective area between filaments in the curved surface of hemisphere- and capsule-shaped formulations, compared with that in the flat surface of the cylinder-shaped formulations. Thus, it was easy for gastric fluid to penetrate into the hemisphere- and capsule-shaped formulations resulting in rapid expanding and the rupture of the formulation. Moreover, the rupture of the hemisphere-shaped formulations was found more quickly than that of the capsule-shaped formulations. The reason was assumed for the poorer axial symmetry of the hemisphere.

The floating time of capsule-0 and capsule-15 was 5.03 ± 0.03 h and 5.28 ± 0.06 h, and that of hemisphere-0 and hemisphere-15 was 4.24 ± 0.19 h and 4.55 ± 0.42 h, respectively. The result indicated that when comparing the same shape of the formulations, those with a higher infill percentage had a slightly longer floating time.

### 3.7. Drug Content

The average drug loading of the drug-loaded filaments was measured to be 9.90 ± 0.05% (*w*/*w*). Drug content of the formulations is shown in Table 2. The drug contents of drug-loaded filaments and printed formulations were between 99.1 to 102.2% of the drugs added in the preparation, indicating that there was no drug loss in either the HME or FDM 3D-printing processes.

### 3.8. In Vitro Drug Release

**Figure 10:**
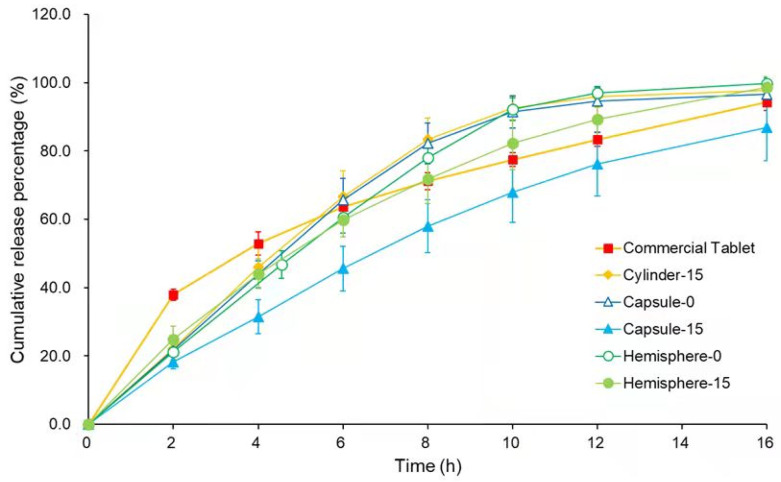
In vitro drug release profiles of verapamil hydrochloride commercial tablet and FDM 3D-printed formulations (*n* = 6).

The in vitro drug release result is shown in Figure 10. Compared with the commercial verapamil hydrochloride tablets, all the 3D-printed formulations achieved a similarly sustained release. The time of 80% drug release was 8 h, 8 h, 16 h, 8 h and 10 h for cylinder-15, capsule-0, capsule-15, hemisphere-0 and hemsphere-15 formulations, respectively. The results demonstrated that the drug-loaded filaments prepared in this study were suitable for manufacturing sustained-release formulations.

The surface area of the three kinds of formulations (shown in Table 1) was designed close to each other in order to ensure a similar rate for water penetrating into the formulations. The results of drug release revealed that the formulations with higher weight released the drug more slowly. Capsule-15 was the heaviest formulation and showed the slowest drug release rate. The weight of cylinder-15, capsule-0 and hemisphere-0 was close, and their release profiles were almost overlapping (*p* > 0.05). In addition, the formulations with higher infill percentages showed slower release rates when comparing capsule-0 with capsule-15 or hemisphere-0 with hemsphere-15, which was consistent with the results reported in literatures [51,52].

As the 3D-printed formulations tended to stick on the paddles (in USP-II apparatus) and prolong the floating time in the drug release test, USP-I apparatus was used in this study [16]. To ensure the sufficient contact of formulations to medium, several dissolution methods were developed and reported [53]. Regarding the influence of in vivo gastric physiology (i.e., gastric motility, transit times, etc.) to the 3D-printed formulations, physiological parameters in the dissolution test, such as fluid volume, pH, agitation speed and so on, should be considered in the future work [54].

## 4. Conclusions

The drug-loaded HME filament composed of HPMC, Soluplus^®^, PEG 400 and verapamil hydrochloride was newly developed and proved highly reliable for FDM 3D-printing sustained-release formulations. The gastric-floating cylinder-, capsule- and hemisphere-shaped formulations with low infill percentage (0% and 15%) were carefully designed and successfully fabricated through FDM 3D-printing technology. The 3D-printed gastric-floating formulations showed desired shapes, elegant appearance, suitable mechanical strength, and good constancy in both formulation weight and drug content. All the formulations floated successfully in vitro with no floating lag time, and released the drug sustainedly for more than 8 h. All the components were thermally stable throughout the whole preparation process and the verapamil hydrochloride was dispersed in an amorphous state, or molecularly. The formulations presented higher mechanical strength when the testing force was added vertically to the orientation of filaments or axial symmetrically to the formulations. The floating time of the formulations depended on their ability to keep intact (not rupture), which was related to the external shape of the formulations. As the surface area of the formulations was designed to be similar, the drug release rate was mainly associated with the weight of the formulations. Moreover, this work demonstrated the potential of preparing FDM 3D-printed gastric floating beyond traditional formulation shapes and infill percentages, to control floating behavior and drug release.

## Figures and Tables

**Table 1:** The parameters of the FDM 3D-printed formulations.

Formulation	Size (mm)		Infill Percentage (%)	Layer Height (mm)	Shell Number	Surface Area (mm^2^)	Volume (cm^3^)	Weight (mg)	Density (g/cm^3^)
	a ^1^	b ^1^							
Cylinder-15	12.0	4.0	15	0.2	3	377.0	0.452	373.5 ± 9.8	0.827
Capsule-0	6.8	17.7	0	0.2	3	377.7	0.560	372.3 ± 17.5	0.664
Capsule-15	6.8	17.7	15	0.2	3	377.7	0.560	404.5 ± 10.3	0.723
Hemisphere-0	11.4	7.8	0	0.2	4	365.1	0.588	374.5 ± 18.8	0.638
Hemisphere-15	11.4	7.8	15	0.2	4	365.1	0.588	389.7 ± 10.1	0.663

**^1^**: a and b are shown in Figure 1.

**Table 2:** In vitro characterization results of formulations.

Formulation	Hardness (*n*)		Floating Time (h)	Drug Content (% *w*/*w*)
	Direction A *	Direction B *		
Cylinder-15	>800	344.8 ± 39.0	6.13 ± 0.50	10.07 ± 0.06
Capsule-0	161.7 ± 7.4	158.6 ± 12.9	5.03 ± 0.33	10.22 ± 0.04
Capsule-15	193.6 ± 28.0	178.3 ± 6.2	5.28 ± 0.06	9.91 ± 0.06
Hemisphere-0	475.7 ± 55.0	78.2 ± 13.1	4.24 ± 0.19	10.13 ± 0.05
Hemisphere-15	505.1 ± 48.4	90.9 ± 16.8	4.55 ± 0.42	10.04 ± 0.15

*: *p* < 0.05.

## Data Availability

Not applicable.

## Supplementary Materials

The following supporting information can be downloaded at: https://www.mdpi.com/article/10.3390/pharmaceutics14020281/s1, Figure S1: DSC results of verapamil hydrochloride. Figure S2: DSC results of PEG 400.

## References

[1] Ahadian S., Finbloom J.A., Mofidfar M., Diltemiz S.E., Nasrollahi F., Davoodi E., Hosseini V., Mylonaki I., Sangabathuni S., Montazerian H. (2020). Micro and nanoscale technologies in oral drug delivery. Adv. Drug Deliv. Rev..

[2] Prinderre P., Sauzet C., Fuxen C. (2011). Advances in gastro retentive drug-delivery systems. Opin. Drug Deliv..

[3] Arora S., Ali J., Ahuja A., Khar R.K., Baboota S. (2005). Floating drug delivery systems: A review. AAPS PharmSciTech.

[4] Lopes C.M., Bettencourt C., Rossi A., Buttini F., Barata P. (2016). Overview on gastroretentive drug delivery systems for improving drug bioavailability. Int. J. Pharm..

[5] Kaushik A.Y., Tiwari A.K., Gaur A. (2015). Role of excipients and polymeric advancements in preparation of floating drug delivery systems. Int. J. Pharm. Investig..

[6] Streubel A., Siepmann J., Bodmeier R. (2006). Gastroretentive drug delivery systems. Expert Opin. Drug Deliv..

[7] Hsiao W.K., Lorber B., Reitsamer H., Khinast J. (2018). 3D printing of oral drugs: A new reality or hype?. Expert Opin. Drug. Deliv..

[8] Norman J., Madurawe R.D., Moore C.M., Khan M.A., Khairuzzaman A. (2017). A new chapter in pharmaceutical manufacturing: 3D-printed drug products. Adv. Drug Deliv. Rev..

[9] Chai X., Chai H., Wang X., Yang J., Li J., Zhao Y., Cai W., Tao T., Xiang X. (2017). Fused deposition modeling (FDM) 3D printed tablets for intragastric floating delivery of domperidone. Sci. Rep..

[10] Chen D., Xu X.Y., Li R., Zang G.A., Zhang Y., Wang M.R., Xiong M.F., Xu J.R., Wang T., Fu H. (2019). Preparation and in vitro evaluation of FDM 3D-printed ellipsoid-shaped gastric floating tablets with low infill percentages. AAPS PharmSciTech.

[11] Ilyés K., Balogh A., Casian T., Igricz T., Borbás E., Démuth B., Vass P., Menyhárt L., Kovács N.K., Marosi G. (2019). 3D floating tablets: Appropriate 3D design from the perspective of different in vitro dissolution testing methodologies. Int. J. Pharm..

[12] Li Q., Guan X., Cui M., Zhu Z., Chen K., Wen H., Jia D., Hou J., Xu W., Yang X. (2018). Preparation and investigation of novel gastro-floating tablets with 3D extrusion-based printing. Int. J. Pharm..

[13] Chen P., Liu J., Zhang K. (2021). Preparation of clarithromycin floating core-shell systems (CSS) using multi-nozzle semi-solid extrusion-based 3D printing. Int. J. Pharm..

[14] Wen H., He B., Wang H., Chen F., Li P., Cui M., Li Q., Pan W., Yang X. (2019). Structure-based gastro-retentive and controlled-release drug delivery with novel 3D printing. AAPS PharmSciTech.

[15] Chen P., Luo H., Huang S., Liu J., Lin M., Yang F., Ban J., Huang Z., Lu Z., Xie Q. (2021). Preparation of high-drug-loaded clarithromycin gastric-floating sustained-release tablets using 3D printing. AAPS PharmSciTech.

[16] Fu J., Yin H., Yu X., Xie C., Jiang H., Jin Y., Sheng F. (2018). Combination of 3D printing technologies and compressed tablets for preparation of riboflavin floating tablet-in-device (TiD) systems. Int. J. Pharm..

[17] Shin S., Kim T.H., Jeong S.W., Chung S.E., Lee D.Y., Kim D.H., Shin B.S. (2019). Development of a gastroretentive delivery system for acyclovir by 3D printing technology and its in vivo pharmacokinetic evaluation in Beagle dogs. PLoS ONE.

[18] Martinez P.R., Goyanes A., Basit A.W., Gaisford S. (2018). Influence of geometry on the drug release profiles of stereolithographic (SLA) 3D-printed tablets. AAPS PharmSciTech.

[19] Khaled S.A., Alexander M.R., Irvine D.J., Wildman R.D., Wallace M.J., Sharpe S., Yoo J., Roberts C.J. (2018). Extrusion 3D printing of paracetamol tablets from a single formulation with tunable release profiles through control of tablet geometry. AAPS PharmSciTech.

[20] McTavish D., Sorkin E.M. (1989). Verapamil. An updated review of its pharmacodynamic and pharmacokinetic properties, and therapeutic use in hypertension. Drugs.

[21] Sawicki W., Głód J. (2004). Preparation of floating pellets with verapamil hydrochloride. Acta Pol. Pharm..

[22] Patel A., Modasiya M., Shah D., Patel V. (2009). Development and in vivo floating behavior of verapamil HCl intragastric floating tablets. AAPS PharmSciTech.

[23] Yoshida M.I., Gomes E.C., Soares C.D., Cunha A.F., Oliveira M.A. (2010). Thermal analysis applied to verapamil hydrochloride characterization in pharmaceutical formulations. Molecules.

[24] Fuenmayor E., Forde M., Healy A.V., Devine D.M., Lyons J.G., McConville C., Major I. (2019). Comparison of fused-filament fabrication to direct compression and injection molding in the manufacture of oral tablets. Int. J. Pharm..

[25] Sahoo J., Murthy P.N., Biswal S., Manik (2009). Formulation of sustained-release dosage form of verapamil hydrochloride by solid dispersion technique using Eudragit RLPO or Kollidon SR. AAPS PharmSciTech.

[26] Kempin W., Domsta V., Grathoff G., Brecht I., Semmling B., Tillmann S., Weitschies W., Seidlitz A. (2018). Immediate release 3D-printed tablets produced via fused deposition modeling of a thermo-sensitive drug. Pharm. Res..

[27] Huang S., O’Donnell K.P., Keen J.M., Rickard M.A., McGinity J.W., Williams R.O. (2016). A new extrudable form of hypromellose: AFFINISOL™ HPMC HME. AAPS PharmSciTech.

[28] Okwuosa T.C., Pereira B.C., Arafat B., Cieszynska M., Isreb A., Alhnan M.A. (2017). Fabricating a shell-core delayed release tablet using dual FDM 3D printing for patient-centred therapy. Pharm. Res..

[29] Goyanes A., Chang H., Sedough D., Hatton G.B., Wang J., Buanz A., Gaisford S., Basit A.W. (2015). Fabrication of controlled-release budesonide tablets via desktop (FDM) 3D printing. Int. J. Pharm..

[30] Okwuosa T.C., Soares C., Gollwitzer V., Habashy R., Timmins P., Alhnan M.A. (2018). On demand manufacturing of patient-specific liquid capsules via co-ordinated 3D printing and liquid dispensing. Eur. J. Pharm. Sci..

[31] Arafat B., Wojsz M., Isreb A., Forbes R.T., Isreb M., Ahmed W., Arafat T., Alhnan M.A. (2018). Tablet fragmentation without a disintegrant: A novel design approach for accelerating disintegration and drug release from 3D printed cellulosic tablets. Eur. J. Pharm. Sci..

[32] Yusif R.M., Abu Hashim I.I., Mohamed E.A., Badria F.A. (2016). Gastroretentive matrix tablets of Boswellia Oleogum resin: Preparation, optimization, in vitro evaluation, and cytoprotective effect on indomethacin-induced gastric ulcer in rabbits. AAPS PharmSciTech.

[33] Qi X., Chen H., Rui Y., Yang F., Ma N., Wu Z. (2015). Floating tablets for controlled release of ofloxacin via compression coating of hydroxypropyl cellulose combined with effervescent agent. Int. J. Pharm..

[34] Maniruzzaman M., Bonnefille M., Aranyos A., Snowden M.J., Douroumis D. (2014). An in-vivo and in-vitro taste masking evaluation of bitter melt-extruded drugs. J. Pharm. Pharmacol..

[35] Nagpal M., Singh S.K., Mishra D. (2013). Superporous hybrid hydrogels based on polyacrylamide and chitosan: Characterization and in vitro drug release. Int. J. Pharm. Investig..

[36] Sawicki W., Łunio R., Walentynowicz O., Kubasik-Juraniec J. (2007). Influence of the type of cellulose on properties of multi-unit target releasing in stomach dosage form with verapamil hydrochloride. Acta Pol. Pharm..

[37] Lunio R., Sawicki W. (2006). Influence of acrylic esters and methacyrlic esters on flotation of pellets and release rate of verapamil hydrochloride. Acta Pol. Pharm..

[38] Gupta S.S., Solanki N., Serajuddin A.T. (2016). Investigation of thermal and viscoelastic properties of polymers relevant to hot melt extrusion, IV: Affinisol™ HPMC HME polymers. AAPS PharmSciTech.

[39] Penumetcha S.S., Gutta L.N., Dhanala H., Yamili S., Challa S., Rudraraju S., Rudraraju S., Rudraraju V. (2016). Hot melt extruded aprepitant-Soluplus solid dispersion: Preformulation considerations, stability and in vitro study. Drug Dev. Ind. Pharm..

[40] Alhijjaj M., Belton P., Qi S. (2016). An investigation into the use of polymer blends to improve the printability of and regulate drug release from pharmaceutical solid dispersions prepared via fused deposition modeling (FDM) 3D printing. Eur. J. Pharm. Biopharm..

[41] Palekar S., Nukala P.K., Mishra S.M., Kipping T., Patel K. (2019). Application of 3D printing technology and quality by design approach for development of age-appropriate pediatric formulation of baclofen. Int. J. Pharm..

[42] Gioumouxouzis C.I., Baklavaridis A., Katsamenis O.L., Markopoulou C.K., Bouropoulos N., Tzetzis D., Fatouros D.G. (2018). A 3D printed bilayer oral solid dosage form combining metformin for prolonged and glimepiride for immediate drug delivery. Eur J. Pharm. Sci..

[43] Goyanes A., Robles Martinez P., Buanz A., Basit A.W., Gaisford S. (2015). Effect of geometry on drug release from 3D printed tablets. Int. J. Pharm..

[44] Dürig T., Fassihi R. (2000). Evaluation of floating and sticking extended release delivery systems: An unconventional dissolution test. J. Control. Release.

[45] Jagdale S.C., Agavekar A.J., Pandya S.V., Kuchekar B.S., Chabukswar A.R. (2009). Formulation and evaluation of gastroretentive drug delivery system of propranolol hydrochloride. AAPS PharmSciTech.

[46] Yom-Tov O., Seliktar D., Bianco-Peled H. (2015). A modified emulsion gelation technique to improve buoyancy of hydrogel tablets for floating drug delivery systems. Mater. Sci. Eng. C Mater. Biol. Appl..

[47] Li Q., Wen H., Jia D., Guan X., Pan H., Yang Y., Yu S., Zhu Z., Xiang R., Pan W. (2017). Preparation and investigation of controlled-release glipizide novel oral device with three-dimensional printing. Int. J. Pharm..

[48] Zhang J., Thakkar R., Zhang Y., Maniruzzaman M. (2020). Structure-function correlation and personalized 3D printed tablets using a quality by design (QbD) approach. Int. J. Pharm..

[49] Kotreka U.K., Adeyeye M.C. (2011). Gastroretentive floating drug-delivery systems: A critical review. Crit. Rev. Ther. Drug Carr. Syst..

[50] Vrettos N.N., Roberts C.J., Zhu Z. (2021). Gastroretentive technologies in tandem with controlled-release strategies: A potent answer to oral drug bioavailability and patient compliance implications. Pharmaceutics.

[51] Aho J., Van Renterghem J., Arnfast L., De Beer T., Rantanen J. (2017). The flow properties and presence of crystals in drug-polymer mixtures: Rheological investigation combined with light microscopy. Int. J. Pharm..

[52] Wu G., Wu W., Zheng Q., Li J., Zhou J., Hu Z. (2014). Experimental study of PLLA/INH slow release implant fabricated by three dimensional printing technique and drug release characteristics in vitro. Biomed. Eng. Online.

[53] Smith D., Kapoor Y., Hermans A., Nofsinger R., Kesisoglou F., Gustafson T.P., Procopio A. (2018). 3D printed capsules for quantitative regional absorption studies in the GI tract. Int. J. Pharm..

[54] Schneider F., Koziolek M., Weitschies W. (2019). In vitro and in vivo test methods for the evaluation of gastroretentive dosage forms. Pharmaceutics.

