# Supplementary material for: FDM 3D-Printed Sustained-Release Gastric-Floating Verapamil Hydrochloride Formulations with Cylinder, Capsule and Hemisphere Shapes, and Low Infill Percentage
Source: Pharmaceutics. 2022 Jan 25;14(2):281. doi: 10.3390/pharmaceutics14020281 (PMC8878517; doi:10.3390/pharmaceutics14020281)
Supplement: Supplementary file 1 [file pharmaceutics-14-00281-s001.zip › pharmaceutics-1521304-supplementary.pdf]

pharmaceutics

Supplementary Materials: FDM 3D-Printed Sustained-Release Gastric-Floating Verapamil Hydrochloride Formulations with Cylinder, Capsule and Hemisphere Shapes, and Low Infill Percentage

Haonan Qian, Di Chen, Xiangyu Xu, Rui Li, Guangrong Yan and Tianyuan Fan

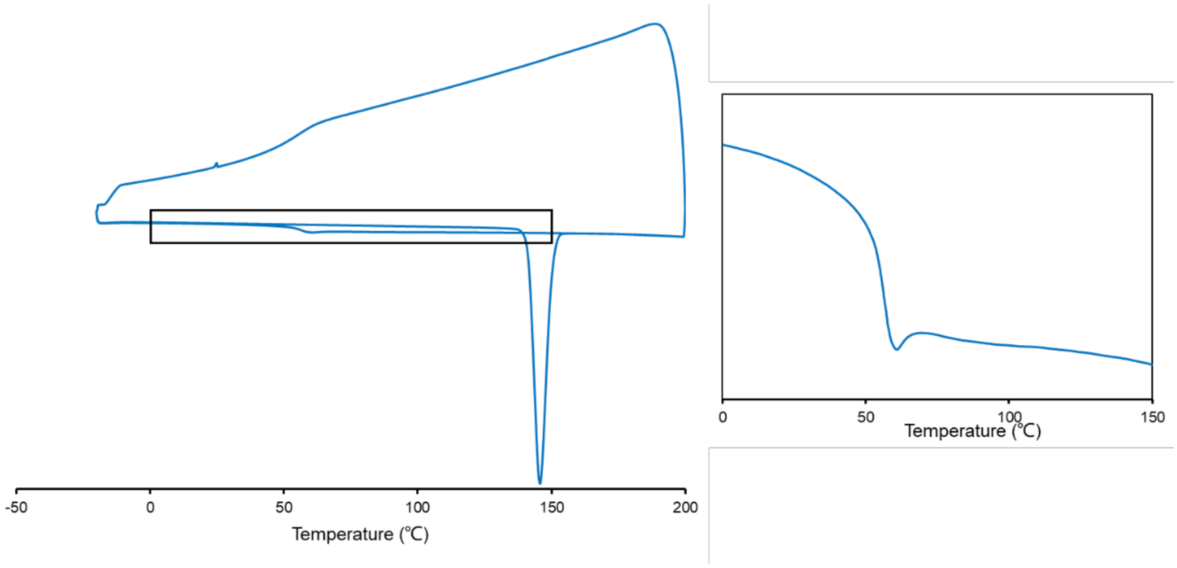

Figure S1. DSC results of verapamil hydrochloride.

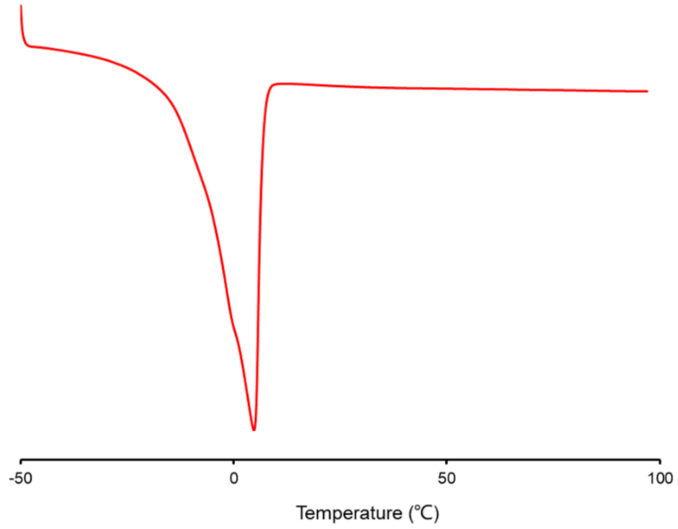

Figure S2. DSC results of PEG 400.
